# Correlates of psychiatric inpatient admission in a paediatric eating disorder cohort

**DOI:** 10.1186/2050-2974-2-S1-O56

**Published:** 2014-11-24

**Authors:** Matthew Hamilton, Hunna Watson, Sarah Egan, Kimberley Hoiles, Emily Harper, Julie McCormack, David Forbes, Chloe Shu

**Affiliations:** 1 Eating Disorders Program, Specialized Child and Adolescent Mental Health Service, Child and Adolescent Health Service, Australia; 2 UNC Center of Excellence for Eating Disorders, University of North Carolina, Chapel Hill, USA; 3 School of Paediatrics and Child Health, Faculty of Medicine, Dentistry and Health Sciences, The University of Western Australia, Perth, Australia; 4 School of Psychology and Speech Pathology, Division of Health Sciences, Curtin University, Perth, Australia

## Objective

The prevalence and correlates of impending psychiatric inpatient admissions in children and adolescents with eating disorders were examined.

## Method

The sample comprised patients aged 8 to 17 years (91% female), with DSM-5 eating disorder diagnosis, categorised as with (n = 38) or without (n = 247) impending psychiatric admission, assessed between 2006 and 2013. The data source was the Helping to Outline Paediatric Eating Disorders (HOPE) Project registry (N ~ 1000), a prospective, ongoing registry study comprising consecutive paediatric tertiary eating disorder referrals.

## Results

Multivariate analysis of variance and discriminant function analysis were conducted to examine correlates. The prevalence of impending psychiatric admission was 13.3%. Significant group differences were found on psychological, behavioural, and situational correlates. Specifically, suicidal ideation, depressive symptoms, eating pathology, multiple methods of weight control, anxiety, purging behaviours, family functioning, and exercise for shape and weight control.

## Conclusions

Almost 1 in 7 young people with an eating disorder who attended assessment had a presentation needing inpatient psychiatric care, and these individuals could be differentiated from individuals not hospitalised or treated in inpatient medical settings. Implications of these findings include better identification of patients at critical psychiatric risk, earlier recognition and intervention for these patients and more focused assessment of comorbid psychiatric symptoms in specialised eating disorder triage and assessment. Adaptions at the study site to clinical and training protocols will be discussed.

This abstract was presented in the **Service Initiatives: Child and Adolescent** stream of the 2014 ANZAED Conference.

